# Diffuse Pigmented Villonodular Synovitis of the Subtalar Joint

**DOI:** 10.5334/jbsr.1477

**Published:** 2018-01-31

**Authors:** Pierre Tritschler, Vincent Baudrez, Eugène Mutijima

**Affiliations:** 1 Centre Hospitalier Régional de Namur, BE; 2 Centre Hospitalier Universitaire, Liège, BE

**Keywords:** MRI, PVNS, diffuse pigmented villonodular synovitis, ankle

A 20-year-old man was referred to our radiology department by his general practitioner for ankle swelling for the prior two months. No injury or trauma was known. Clinical examination showed swollen ankle without frank pain or cutaneous inflammation. The patient had no previous medical or surgical history. Magnetic Resonance Imaging (MRI) of the right ankle was performed.

Sagittal T1-weighted images without and with gadolinium injection show an intra-articular hypointense mass (Figure 1a, white star) in the subtalar joint with heterogeneous enhancement (Figure 1b, black star).

**Figure 1:**
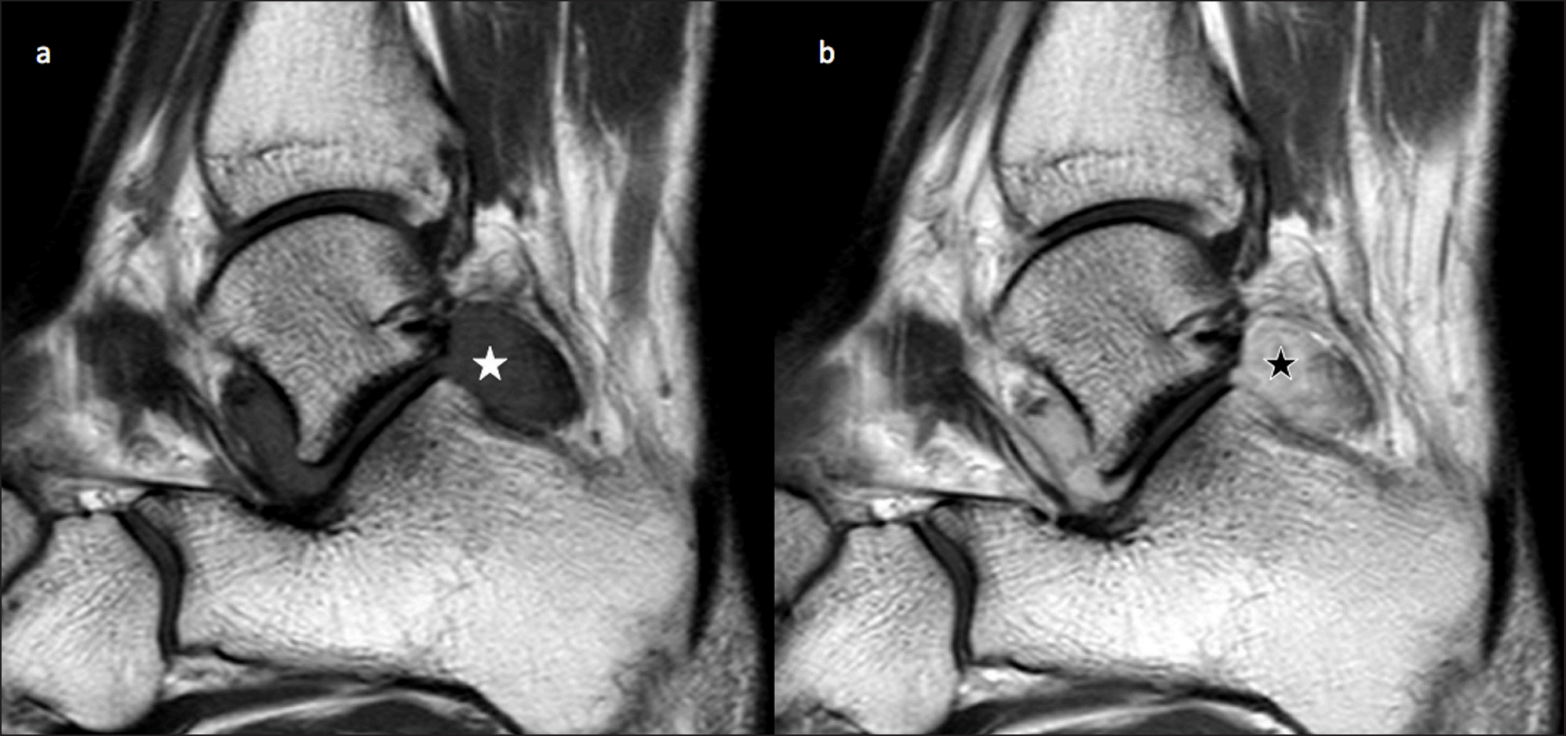
**a.** Sagittal T1-weighted image without gadolinium injection shows an intra-articular hypointense mass (white star) in the subtalar joint. **b.** Sagittal T1-weighted image with gadolinium injection shows the mass with heterogeneous enhancement (black star).

Sagittal Turbo Spin-Echo T2-weighted images show an hypointense mass (Figure 2a, white star) with low signal intensity foci corresponding to hemosiderin deposition (Figure 2a, white arrows). On Gradient Echo T2-weighted images, hemosiderin deposition appears more visible because of the blooming effect due to the magnetic susceptibility (Figure 2b, white arrows). There were no bony erosions. The diagnosis of diffuse pigmented villonodular synovitis (PVNS) of the subtalar joint was suspected. Surgical excision was performed and the histological examination confirmed the diagnosis of PVNS with mononuclear histiocyte-like cells with rare multinucleated giant cells (Figure 3a, black arrows) and hemosiderin-laden macrophages (Figure 3b).

**Figure 2:**
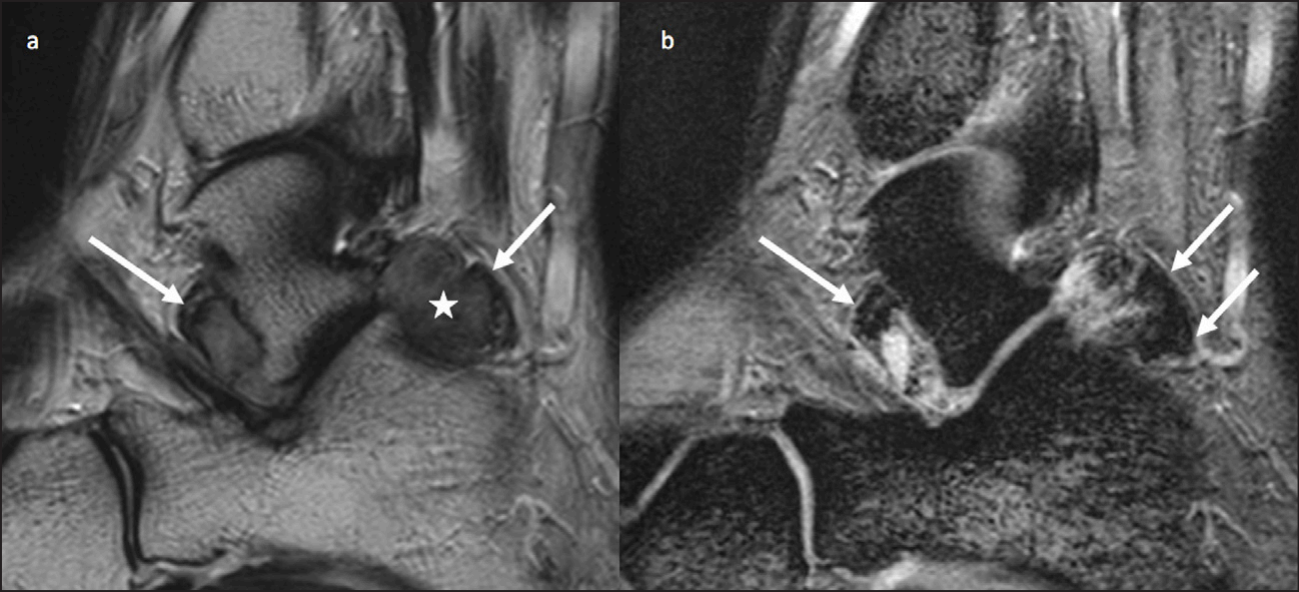
**a.** Sagittal Turbo Spin-Echo T2-weighted image shows an hypointense mass (white star) with low signal intensity foci corresponding to hemosiderin deposition (white arrows). **b.** On Sagittal Gradient Echo T2-weighted images, hemosiderin deposition appears more visible because of the blooming effect due to the magnetic susceptibility (white arrows).

**Figure 3:**
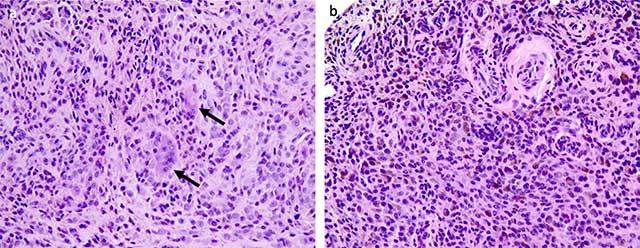
**a.** Photomicrograph (hematoxylin-eosin stain, power field view × 20) shows mononuclear histiocyte-like cells with rare multinucleated giant cells. **b.** Photomicrograph (hematoxylin-eosin stain, power field view × 20) shows hemosiderin-laden macrophages.

## Comment

Pigmented villonodular synovitis (PVNS) is an uncommon proliferative disease with an uncertain etiology, described for the first time in 1941 by Jaffe. PVNS is considered as a benign pathology characterized by focal or diffuse synovial villi hyperplasia with hemosiderin deposition. Few cases of malignant transformation are described.

There are two types of PVNS: localized and diffuse disease with intra-articular or extra-articular involvement. PVNS is a disabling disease with progressive joint destruction. Affected joints in descending order of frequency were knee, hip, ankle, shoulder, and elbow. On plain film radiography and Computed Tomography, PVNS features are non-specific with the presence of joint effusion and soft-tissue swelling. Bony erosions are present in late stages, especially in the joints with small synovial extension as hip and ankle.

Ultrasound demonstrates nonspecific joint effusion and hypoechoic thickening of the synovium. High vascularization is usually seen on Doppler imaging.

MRI is the most effective imaging technique to evaluate this disease. On T1-weighted images, PVNS presents a low to intermediate signal with a variable enhancement after gadolinium injection. On T2-weighted images, PVNS demonstrates a low to intermediate signal and a high signal on STIR imaging. Gradient Echo imaging is an efficient diagnostic tool revealing the disproportionately lower signal intensity of hemosiderin deposition.

Histopathological feature of PVNS is characterized by two types of mononuclear cells, small histiocyte-like cells which represent the main cellular component, and larger cells with abundant cytoplasm, nuclei with reniform or lobulated shape, associated with multinucleated giant cells and hemosiderin-laden macrophages.

Reference treatment is surgery; radiosyniovectomy as adjuvant treatment should be considered depending the case. The recurrence rate of diffuse intra-articular PVNS is variable, ranging from 8% to 56% [1]. To conclude, MRI is an important tool to characterize PVNS.
